# A lattice topology optimization of cervical interbody fusion cage and finite element comparison with ZK60 and Ti-6Al-4V cages

**DOI:** 10.1186/s12891-021-04244-2

**Published:** 2021-04-26

**Authors:** Jun Sun, Qiuan Wang, Dazhao Cai, Wenxiang Gu, Yiming Ma, Yang Sun, Yangyang Wei, Feng Yuan

**Affiliations:** Departments of Orthopedics, The Affiliated Hospital of Xuzhou Medical University, 99 Huaihai West Rd, Xuzhou, 221006 China

**Keywords:** Fusion cage, Cervical spine, Finite element analysis, Biomechanics

## Abstract

**Background:**

In current clinical practice, the most commonly used fusion cage materials are titanium (Ti) alloys. However, titanium alloys are non-degradable and may cause stress shielding. ZK60 is a bio-absorbable implant that can effectively avoid long-term complications, such as stress shielding effects, implant displacement, and foreign body reactions. In this study, we aimed at investigating the biomechanical behavior of the cervical spine after implanting different interbody fusion cages.

**Methods:**

The finite element (FE) models of anterior cervical disc removal and bone graft fusion (ACDF) with a ZK60 cage and a Ti cage were constructed, respectively. Simulations were performed to evaluate their properties of flexion, extension, lateral bending, and axial rotation of the cervical spine. Moreover, a side-by-side comparison was conducted on the range of motion (ROM), the deformation of cages, the stress in the cages, bone grafts, and cage-end plate interface. Simultaneously, according to the biomechanical analysis results, the microporous structure of the ZK60 cage was improved by the lattice topology optimization technology and validation using static structure.

**Results:**

The ROMs in the current study were comparable with the results reported in the literature. There was no significant difference in the deformation of the two cages under various conditions. Moreover, the maximum stress occurred at the rear of the cage in all cases. The cage’s and endplate-cage interface’s stress of the ZK60 group was reduced compared with the Ti cage, while the bone graft stress in the ZK60 fusion cage was significantly greater than that in the Ti fusion cage (average 27.70%). We further optimized the cage by filling it with lattice structures, the volume was decreased by 40%, and validation showed more significant biomechanical properties than ZK60 and Ti cages.

**Conclusion:**

The application of the ZK60 cage can significantly increase the stress stimulation to the bone graft by reducing the stress shielding effect between the two instrumented bodies. We also observed that the stress of the endplate-cage interface decreased as the reduction of the cage’s stiffness, indicating that subsidence is less likely to occur in the cage with lower stiffness. Moreover, we successfully designed a porous cage based on the biomechanical load by lattice optimization.

## Introduction

Cervical spondylosis has become a common clinical disease as people’s living habits change and work pressure elevates. The middle-aged and elderly populations are the leading disease groups of cervical spondylosis, while there is a trend of getting younger in recent years. Intervertebral disc degeneration (IDD), which is also called degenerative disc disease, is the primary cause of degenerative spinal disease and one of the most common ailments severely affecting the quality of life in elderly populations and a series of secondary alternations (such as nucleus pulposus herniation and prolapse, bone spur formation, and secondary spinal stenosis), causing a variety of symptoms and signs. Surgical treatment is available for patients with cervical spondylosis who have failed conservative treatment and whose daily life has been significantly affected by cervical spondylosis. Anterior cervical disc removal and bone graft fusion (ACDF) were firstly proposed by Cloward [1] in the 1950s, which have the advantages of less trauma, thorough decompression, and effective restoration of the cervical spine’s physiological curvature [2, 3]. Bagby first introduced the intervertebral fusion cage technology in 1988 [4], which can avoid long-term pain, infection, bone graft collapse, and immune rejection caused by autologous iliac bone transplantation and xenogeneic bone transplantation. The intervertebral fusion cage technology is widely used to provide stability during vertebral body movement and bone fusion [5]. In current clinical practice, the most commonly used fusion cage materials are titanium (Ti) alloys and polyetheretherketone (PEEK) [6–10]. Titanium has good corrosion resistance and excellent mechanical properties [7–9]. However, titanium alloys are non-degradable and belong to biologically inert materials, permanently remaining after implantation, thereby increasing the implant’s risk of breakage. The titanium alloy’s elastic modulus is 110 GPa, much higher than that of human cortical bone (18 GPa) [11]. A higher elastic modulus would cause a more considerable stress difference between the newly formed bone and the cage, resulting in the interface relaxation and the formation of the stress shielding layer, which is not conducive to the growth of new bone. Simultaneously, the force on the contact surface between the endplate and the cage would also elevate, increasing the probability of postoperative cage settlement.

Therefore, discovering a degradable material with properties similar to bones has become an attractive research area. Bio-absorbable implants can effectively prevent long-term complications, such as stress shielding effects, implant displacement, and foreign body reactions [12]. Besides, since the degradation space can be continuously replaced by cancellous bone, a complete intervertebral fusion can be achieved. As an orthopedic implant material, the density and elastic modulus of Magnesium (Mg) alloy (1.738 g/cm^3^, 43 GPa) are much closer to normal bone tissue (1.75 g/cm^3^, 18 GPa) than traditional metals (e.g., Ti: 4.47 g/cm^3^, 110 GPa), and the lower stiffness of the cages means less gradual dynamic loading and stress shielding of the fusion site [13]. Magnesium alloy also has relatively high strength and stiffness, bone conduction activity, and radiation permeability relative to titanium alloy, making it a potential cage material [14]. The current research on magnesium alloys used in biomedicine is mainly concentrated on magnesium alloys AZxx (containing aluminum and zinc) and AMxx (containing aluminum and manganese) [15–17]. The biosafety of aluminum content presented in AZxx and AMxx alloys is arguable as aluminum adversely affects osteoblasts and is reported to be neurotoxic [18–20]. Therefore, the aluminum-free magnesium alloy ZK60 was selected as the preferred material for this study. The magnesium alloy ZK60 contains zinc and zirconium as the main alloying elements that are biologically friendly to the human body. Relevant studies have shown that a cage with a pore structure can promote new bone growth [21–23]. It is challenging to manufacture complex porous structures by the traditional casting process, while 3D printing facilitates the manufacturing of porous fusion cages. At present, the mechanical research on magnesium alloy cages mainly focuses on screws and plates. There are still few studies on magnesium alloy cages, and it is unclear if they can effectively avoid the stress shielding effect and provide immediate stability.

In this work, we aimed at investigating the biomechanical behavior of the cervical spine after implanting different interbody fusion cages. Simultaneously, according to the biomechanical analysis results, the microporous structure of the magnesium alloy cage was improved by the lattice topology optimization technology and validation of static structure. The findings may provide new insights into the design and manufacture of cervical fusion cages in the future.

## Methods

### Simulation environment

The simulations were performed on a workstation consists of Windows 10 Pro system, CPU (AMD Ryzen7 3700X), RAM (32 GB), and GPU (Nvidia GeForce RTX 3080). Compute Unified Device Architecture (CUDA) library was installed in the workstation, and GPU was enabled for ANSYS GPU acceleration.

### Construction of the three-dimensional model of the cervical spine

This research was conducted at The Affiliated Hospital of Xuzhou Medical University, Xuzhou, China. The computed tomography (CT) images of the cervical spine were obtained at a 1-mm interval from one of our authors (male; age: 25 years; weight: 76 kg; height: 177 cm) who had never had any cervical disease. The CT images were then imported into Mimics 21 (Materialise, Inc., Belgium). According to the CT gray value, the C2-C5 three-dimensional model was established and imported into 3-Matic (Materialise, Inc., Belgium) software for further processing. The establishment of the intervertebral disc’s soft tissue model and the articular surface and the optimization of the vertebral body were performed in 3-Matic. Both the cortical bone and vertebral endplate were modeled with a thickness of 1 mm. The intervertebral disc was modeled as two distinct regions: the annulus fibrosus and the nucleus pulposus.

### Finite element analysis (FEA) model establishment

**Fig. 1:**
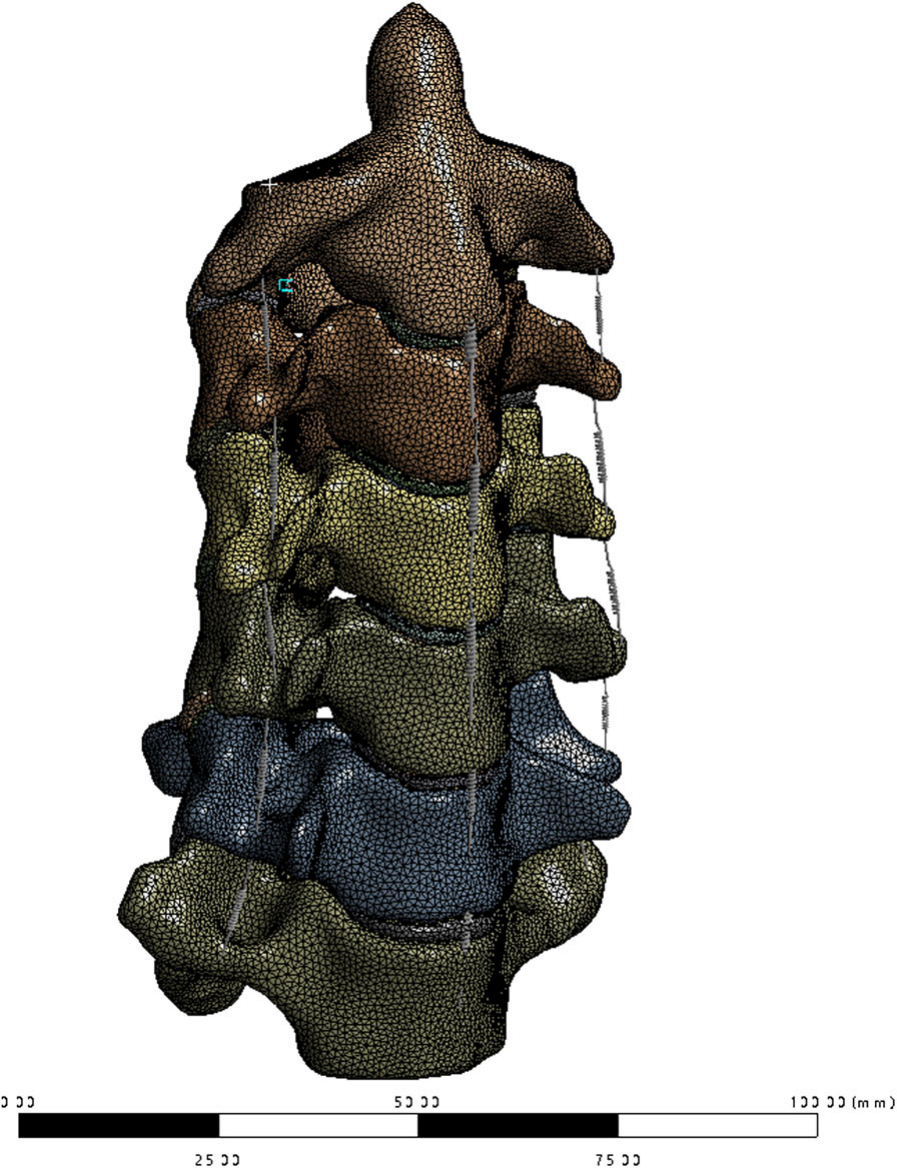
The FE model intact cervical spine

The cortical bone, cancellous bone, posterior bone structure, intervertebral discs, and cartilage were all meshed into Solid192 elements, and then the CDB file was exported into ANSYS 2020R2 (ANSYS, Inc. Delaware, USA). Five major intervertebral ligaments (anterior longitudinal ligament, posterior longitudinal ligaments, flava ligaments, capsular ligaments, and interspinous ligaments) were constructed at corresponding anatomical positions, and the ligaments were defined as spring connections [24] (Fig. 1). The contact between the facet joints was defined as frictional contact with a friction coefficient of 0.1. Other adjacent parts were defined as bonded contact. The properties are shown in Table 1 [25–29].

**Table 1:** Material properties of all components

Component	Density (kg/m3)	Young’s Modulus	Poisson’s Ratio	Stiffness (N/mm)	Type
Cortical bone	1830	12,000	0.29		Solid
Cancellous bone	1000	450	0.29		Solid
Endplate	1830	1000	0.4		Solid
Nucleus	1000	3.4	0.49		Solid
Annulus fibrosus	1200	4.2	0.4		Solid
ZK60	1835	44,660	0.305		Solid
Ti-6Al-4V	4429	113,800	0.339		Solid
Facet joint	1000	10	0.3		Solid
Ligament					
ALL	1100	15	0.3	16	Spring
PLL	1100	10	0.3	25	Spring
CL	1100	4	0.3	19	Spring
ISL	1100	4	0.3	7	Spring
LF	1100	5	0.3	25	Spring

*ALL* Anterior longitudinal ligament, *CL* Cervical ligament, *FL* Flaval ligament, *ISL* Interspinous ligament, *PLL* Posterior longitudinal ligament

FEA model establishment was accomplished using a hybrid loading protocol [30]. Briefly, the original form of this protocol consisted of (a) applying pure moments to intact spine, (b) applying pure moments to implanted spine until its ROM is equal to the ROM of the intact spine (i.e., results from the previous step), and (c) the statistical comparison of the biomechanical variables in the two conditions. This motion-controlled moment loading was selected to simulate the clinical setting related to the total motion of the cervical spine [30–33]. A force of 73.6 N was applied to the upper surface of the C2 vertebral body, 1.5 N/M was applied in the X-axis direction according to the right-hand rule, and a moment of 1.0 N/M was applied to the Y-axis and Z-axis to simulate the flexion, extension, left/right lateral bending, and left/right axial rotation of the cervical spine. Based on the values reported in the literature, the ROM of the complete spine model was verified [34–36].

### Cage modeling and surgical simulations

**Fig. 2:**
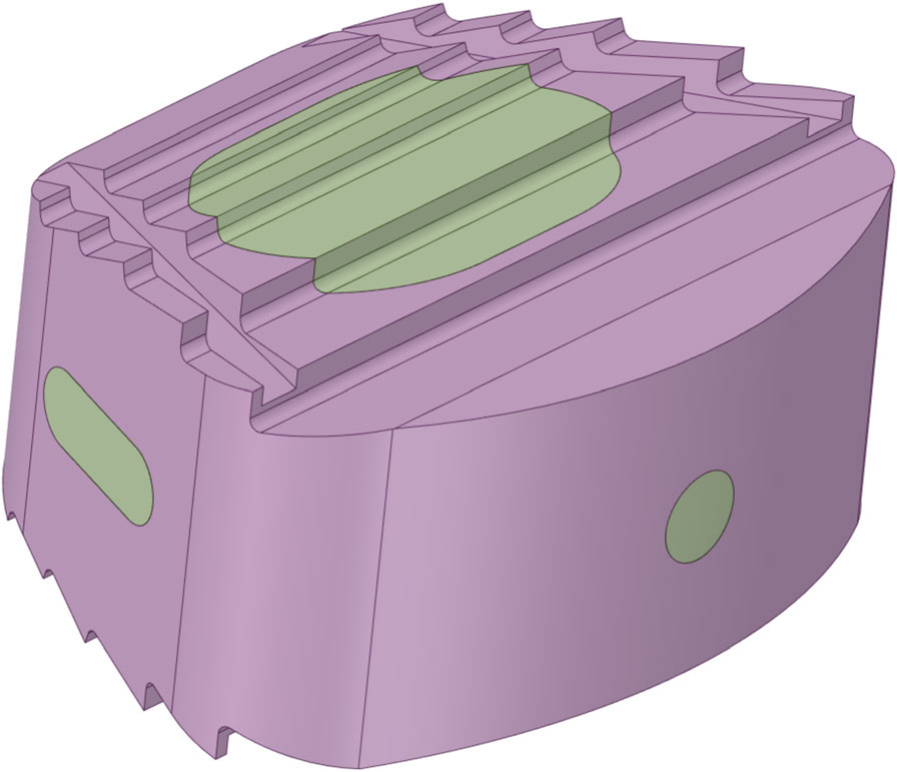
The model of the interbody fusion cage

**Fig. 3:**
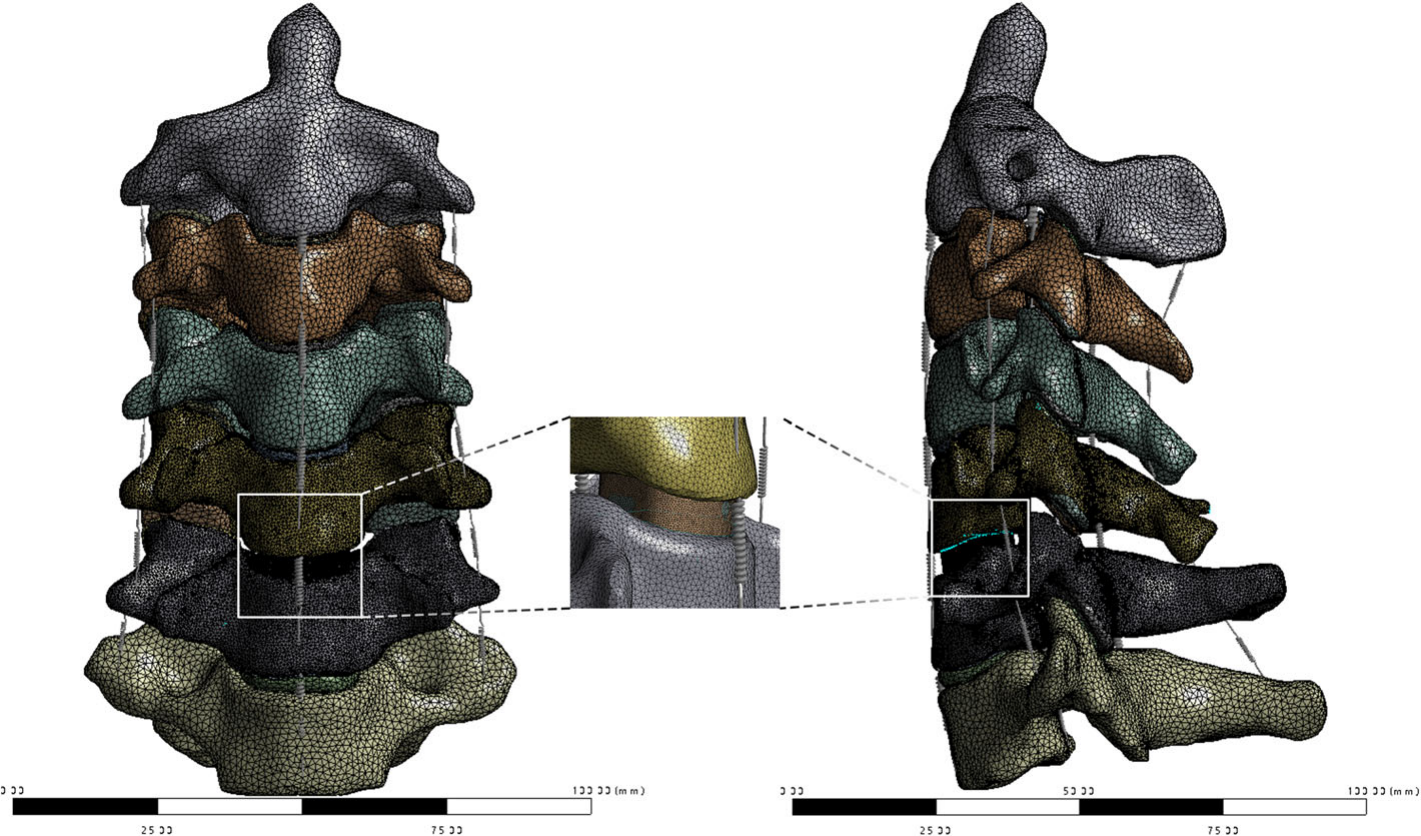
The model of ACDF surgery FE model, the fusion cage was placed in C5/6

The interbody fusion cage was constructed in Solidworks 2020 (Dassault Inc., Concord, USA) and was exported as an STL file (Fig. 2). The cage was defined as ZK60 and Ti-6Al-4V (Ti) to perform interbody fusion simulations. The C2-C7 finite element model was modified on the C5/6 functional spine unit; the C5-6 intervertebral disc was removed; the cervical fusion cage was placed in a suitable position; the fusion cage was filled with cancellous bone material to simulate bone grafting; the interfaces between the cage and bone grafts were bonded (Fig. 3). All motion conditions were independently calculated for both simulation groups, and results were outputted and recorded for further analysis.

### Comparisons of results

The results were collected, including the cervical spine and fusion cage’s deformation, the equivalent stress of cervical spine, fusion cage, bone graft, endplate-cage interface, and adjacent discs. The results of each group under different conditions were recorded and compared.

### Lattice optimization

To optimize the topology of the cage based on the load characteristics without affecting the stability, the target porosity was set to be 40- 60% according to the optimal porosity reported in the previous literature [37–39], and the octet lattice was implemented. The lattice size was set to 2 mm, and the density variable was between 0.3 to 1.0 to prevent a small lattice structure and slow down the cage degradation. The optimization results were exported to SpaceClaim: 3D Modeling Software (ANSYS, Inc. Delaware, USA) for post-processing, shelling out the cage for lattice filling and retaining the 0.5 mm shell to maintain long-term stability.

### Validation of lattice structure

The validation used a homogenization model to simulate the lattice structure. Homogenization was put into the cervical model under all conditions (Optimized), and the results were analyzed and compared with the previous model.

## Results

### Validation of the intact cervical model

**Fig. 4:**
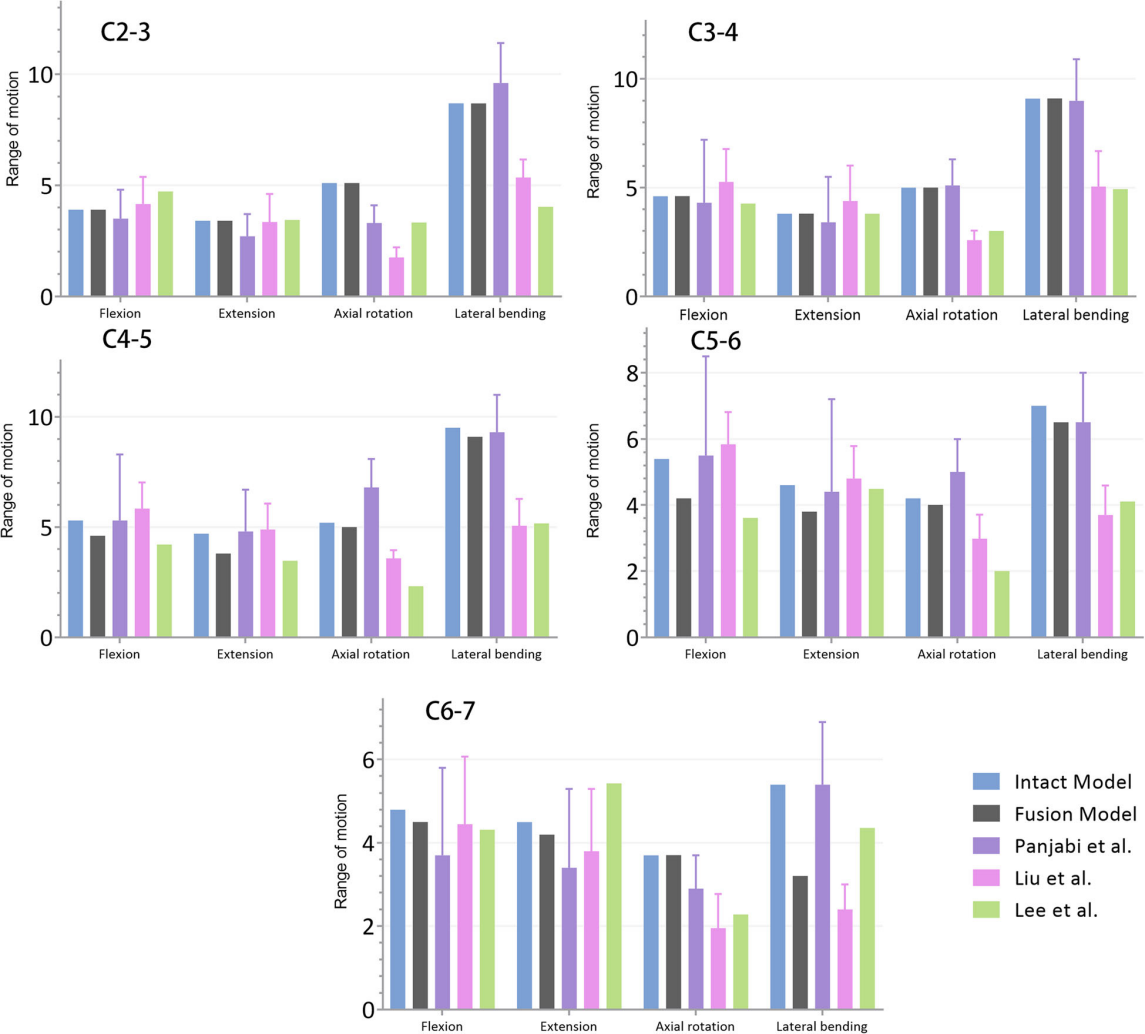
The comparison of ROMs with previous studies under all circumstances

The predicted ROMs for all levels were compared with those in previous biomechanical and finite element analysis studies [34–36]. The ROMs of the intact model under all circumstances are measured and analyzed. The ROMs in the current study showed comparable results with those in previous studies. However, the ROMs of fusion models were not significantly different from the intact model. The comparison of ROMs is shown in Fig. 4.

### Total deformation of the fusion cage

**Fig. 5:**
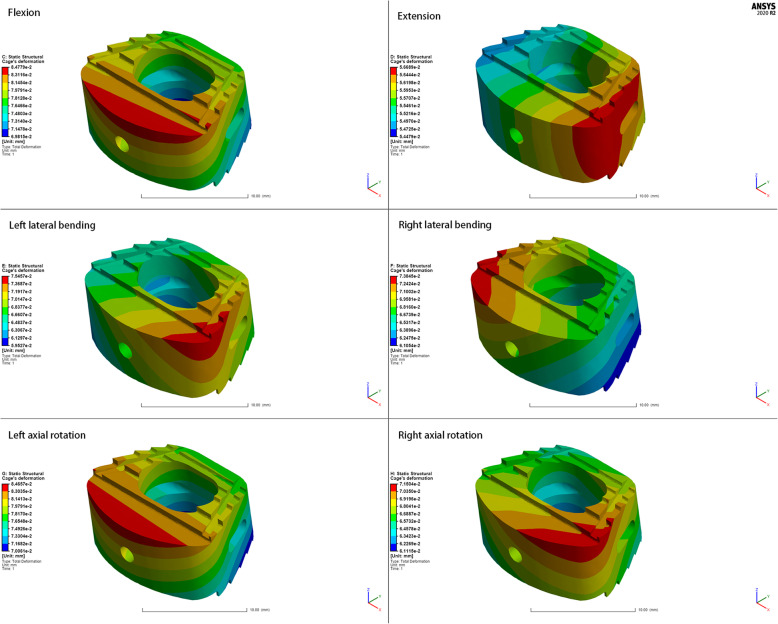
The cloud map of ZK60 cage’s deformation

**Fig. 6:**
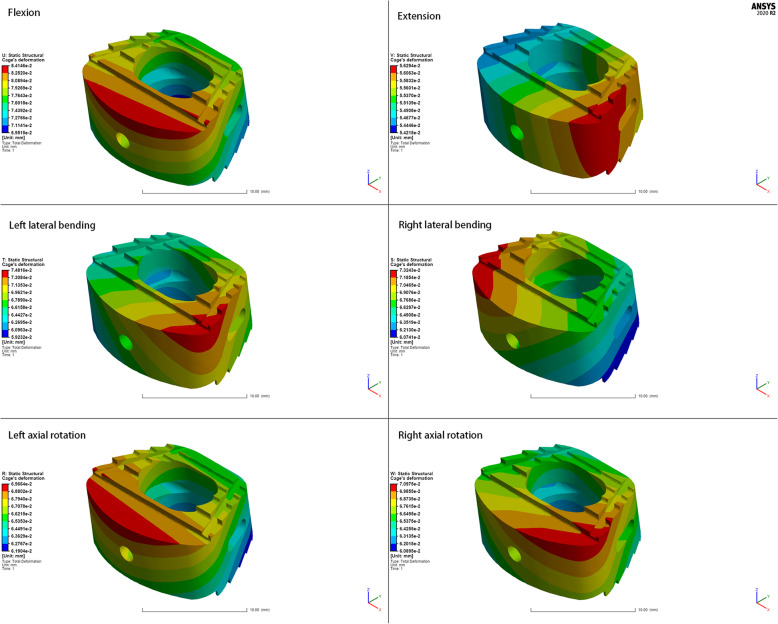
The cloud map of Ti-6Al-4V cage’s deformation

The deformations of the fusion cage shown in Figs. 5 and 6 were calculated under all conditions. Both the ZK60 cage and the titanium alloy cage produced slight deformations. The cage deformation reached the maximum when the cervical spine was flexed (ZK60: 84.78 μm; Ti-6Al-4V: 84.15 μm), while the deformation was the smallest when it was extended (ZK60: 56.68 μm; Ti-6Al-4V: 56.29 μm). Moreover, there was no significant difference in the deformation of the two cages under various conditions. The deformation mainly existed at the front edge of the upper surface of the cage.

### Equivalent stress of fusion cage and bone graft

**Fig. 7:**
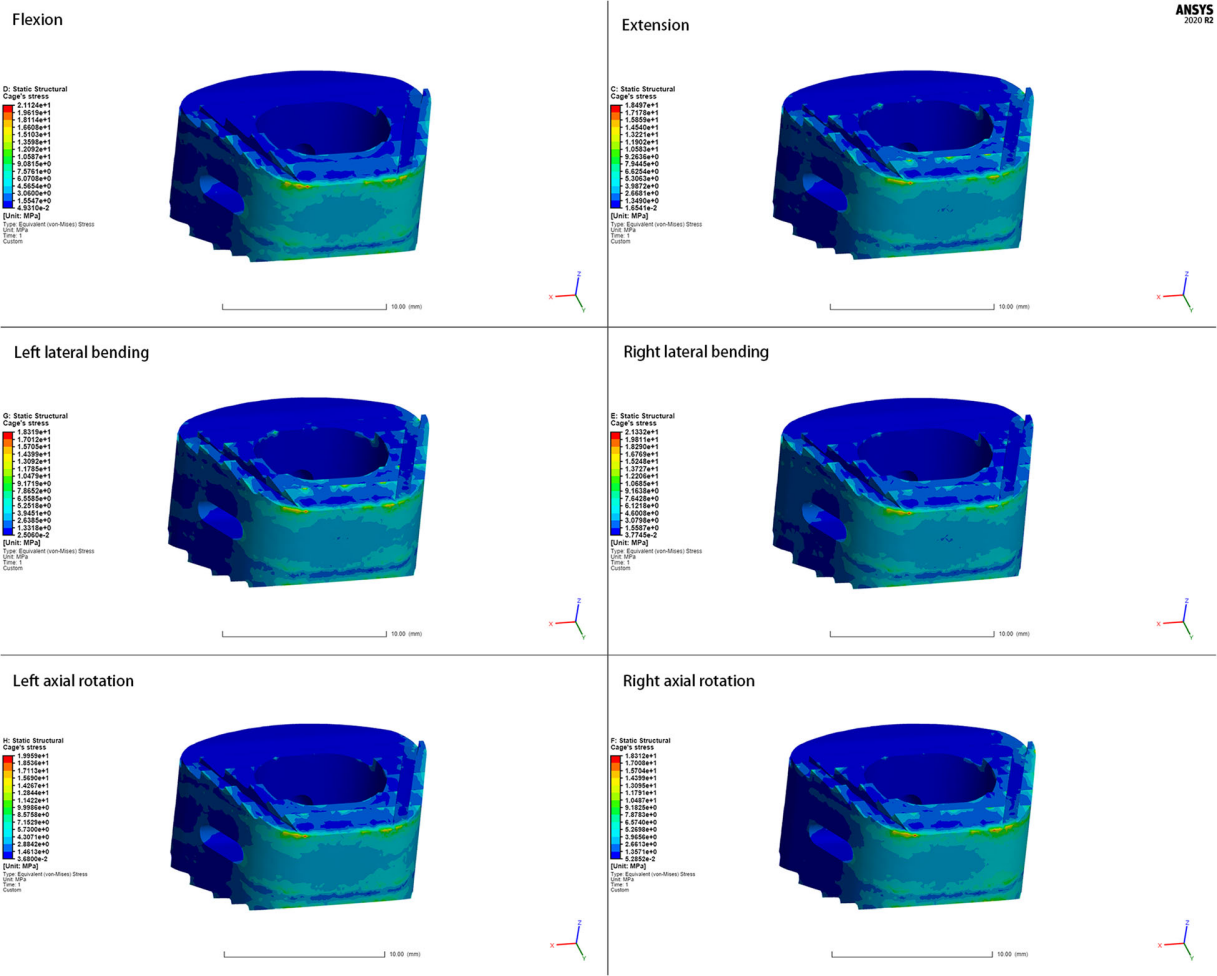
The cloud map of ZK60 cage’s von-mises stress in all motion modes

**Fig. 8:**
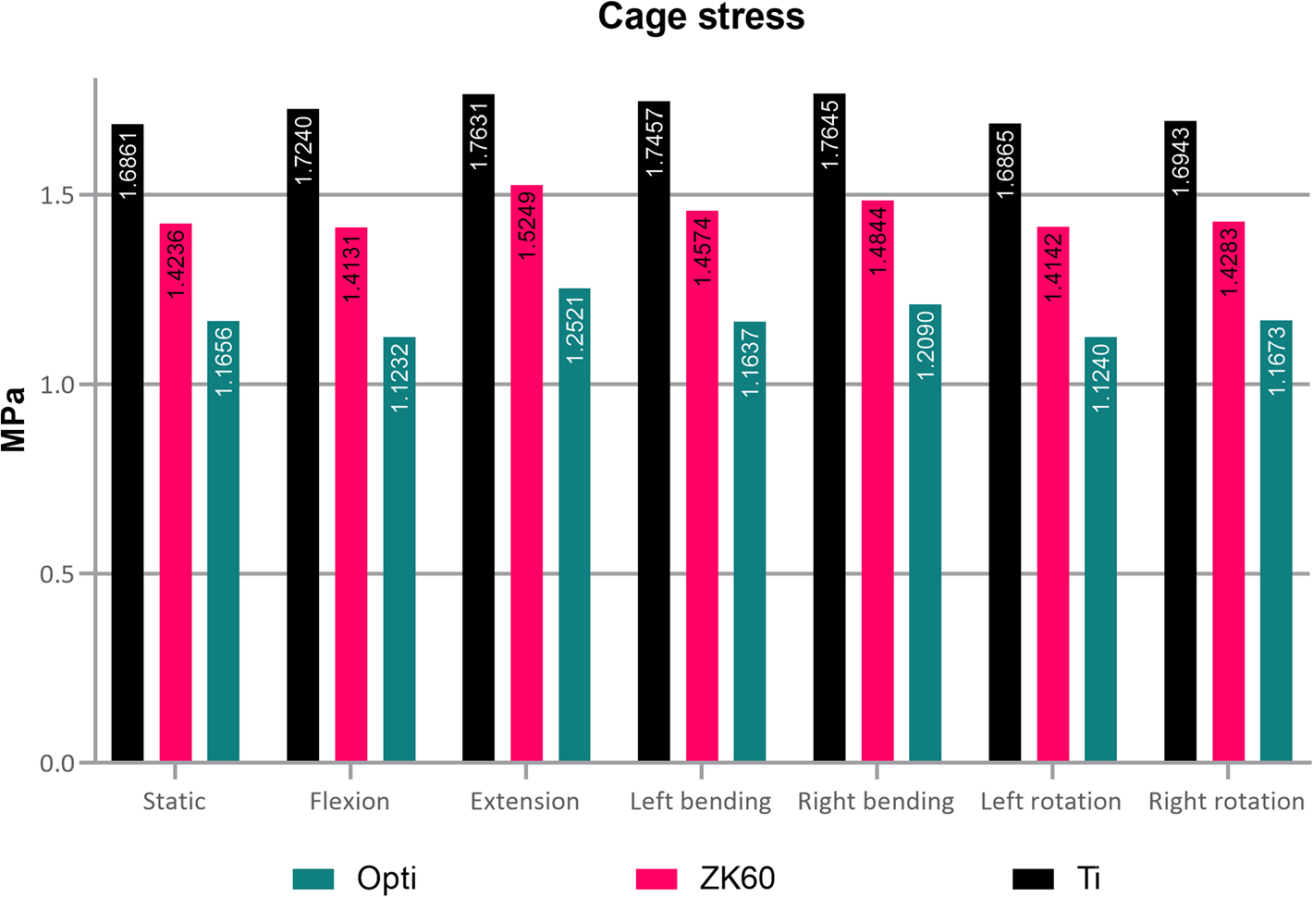
Von-mises stress of various fusion cages under all circumstance

The stress of the ZK60 cage under different conditions is shown in Fig. 7. The maximum stress occurred at the rear of the cage in all cases. The maximum stress for the ZK60 cage was 21.33 MPa, while the maximum for the Ti cage was 32.31 MPa. The stress and difference among the ZK60, optimized, and the Ti cage under all conditions shown in Fig. 8 exhibited that the averaged stress of the ZK60 cage was reduced by about 15.91% compared with the Ti cage (Table 2).

**Table 2:** Equivalent stress of various cages and bone grafts under all circumstances

Equivalent Stress						
	Ti	ZK60	Optimized	ZK60 vs Ti	Optimized vs ZK60	Optimized vs Ti
Cage (MPa)						
Static	1.69	1.42	1.17	−15.57%	−18.12%	−30.87%
Flexion	1.72	1.41	1.12	−18.03%	−20.52%	−34.85%
Extension	1.76	1.52	1.25	−13.51%	−17.89%	−28.98%
Left lateral bending	1.75	1.46	1.16	−16.51%	−20.15%	−33.34%
Right lateral bending	1.76	1.48	1.21	−15.87%	−18.55%	−31.48%
Left axial rotation	1.69	1.41	1.12	−16.15%	−20.52%	−33.35%
Right axial rotation	1.69	1.43	1.17	−15.70%	−18.27%	−31.10%
Bone graft (KPa)						
Static	37.99	48.77	69.03	28.37%	41.55%	81.70%
Flexion	38.42	47.91	67.24	24.69%	40.36%	75.02%
Extension	39.10	51.26	74.79	31.07%	45.91%	91.25%
Left lateral bending	39.00	49.67	70.45	27.38%	50.56%	91.77%
Right lateral bending	38.66	49.48	70.00	27.99%	42.39%	82.24%
Left axial rotation	37.95	47.87	67.12	26.13%	40.21%	76.85%
Right axial rotation	38.14	48.93	69.33	28.29%	41.69%	81.79%

**Fig. 9:**
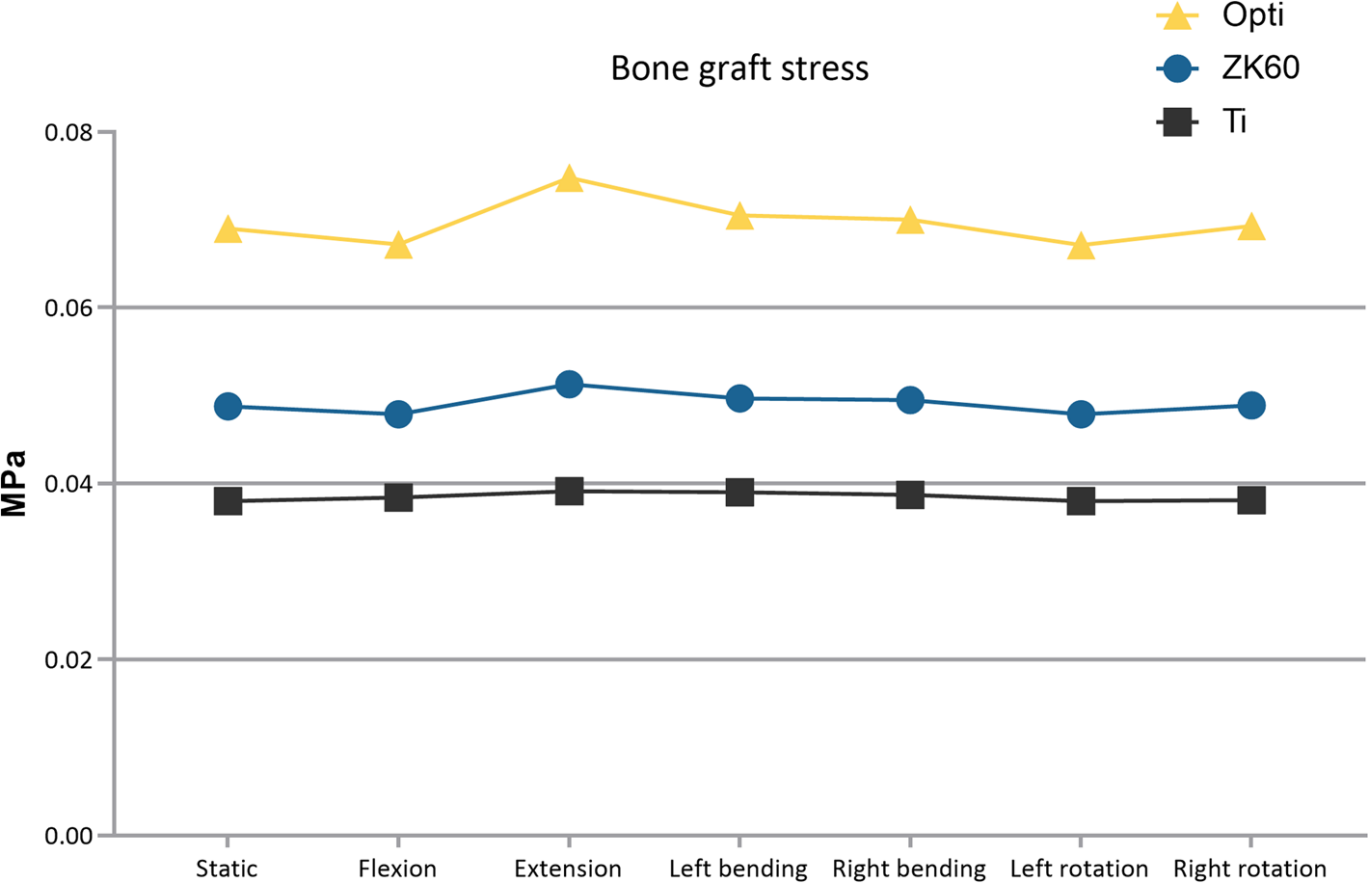
Average stress of bone grafts in various fusion cages

The forces on the bone grafts in the ZK60, optimized, and Ti cages under various loading conditions are summarized in Table 2 and Fig. 9. The bone graft stress in the ZK60 fusion cage was significantly greater than that of the Ti fusion cage (average 27.70%).

### The maximum stress in the endplate-cage interface at the treatment level

**Fig. 10:**
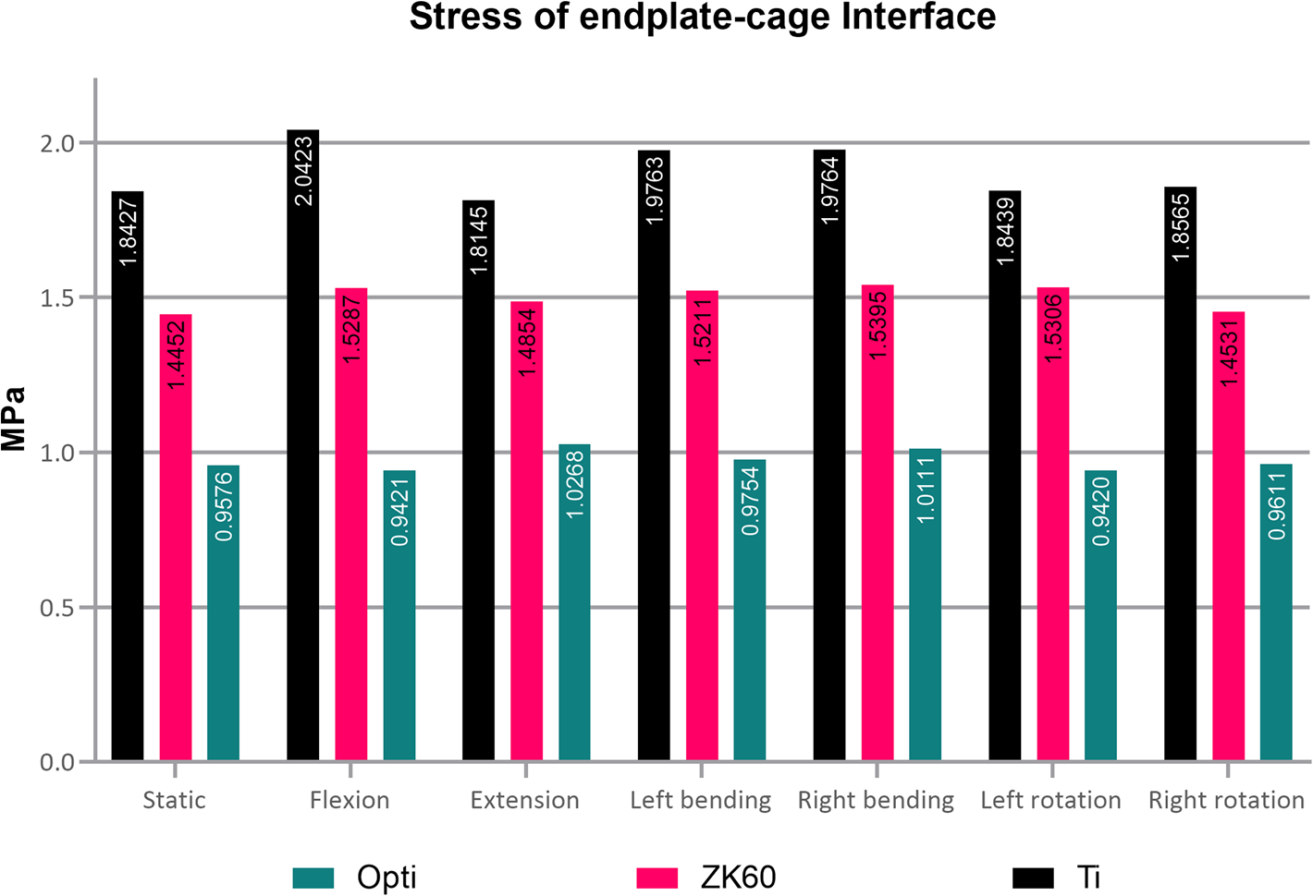
Average stress of endplate-cage interface in various fusion cages

In the study, the interfacial stress of the ZK60 cage was significantly lower relative to that of the Ti group (Fig. 10), which could be inferred based on the analysis that the maximum stress occurred at the rear edge of the upper endplate. In contrast, the stress at the front edge of the final plate was relatively small.

### Lattice optimization according to the biomechanical load

**Fig. 11:**
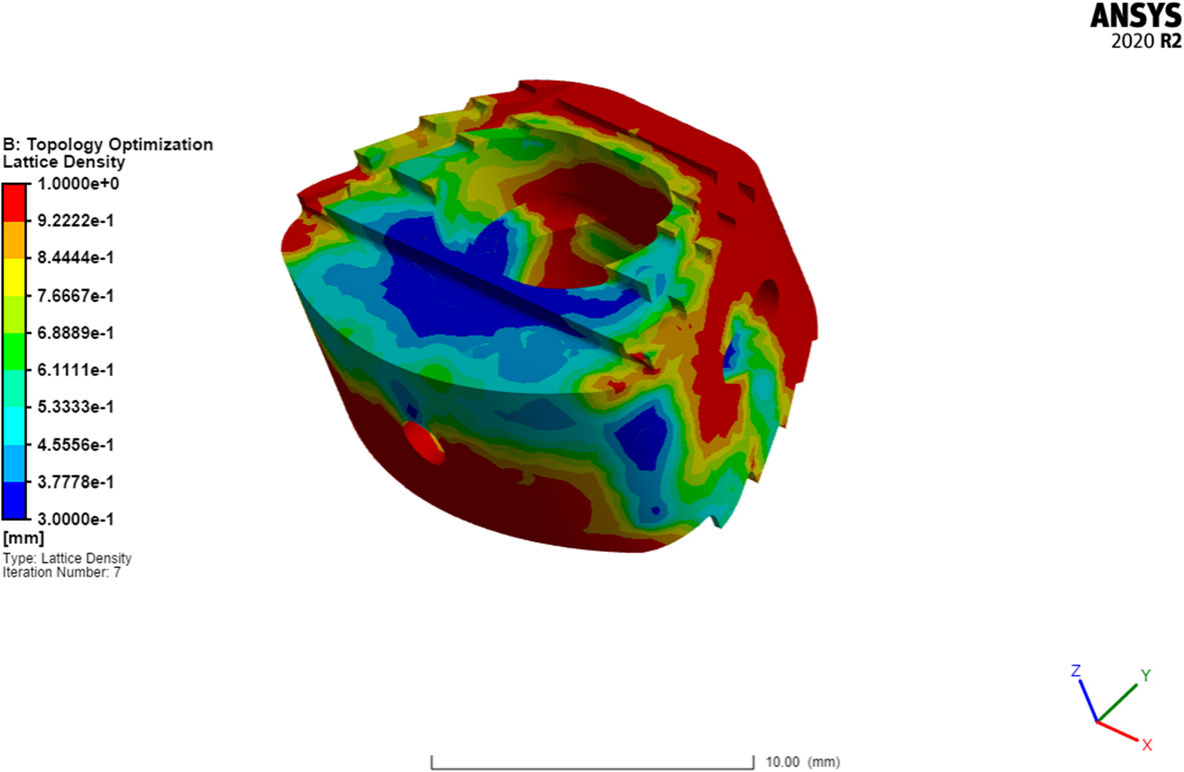
The lattice density cloud map of topology optimization

**Fig. 12:**
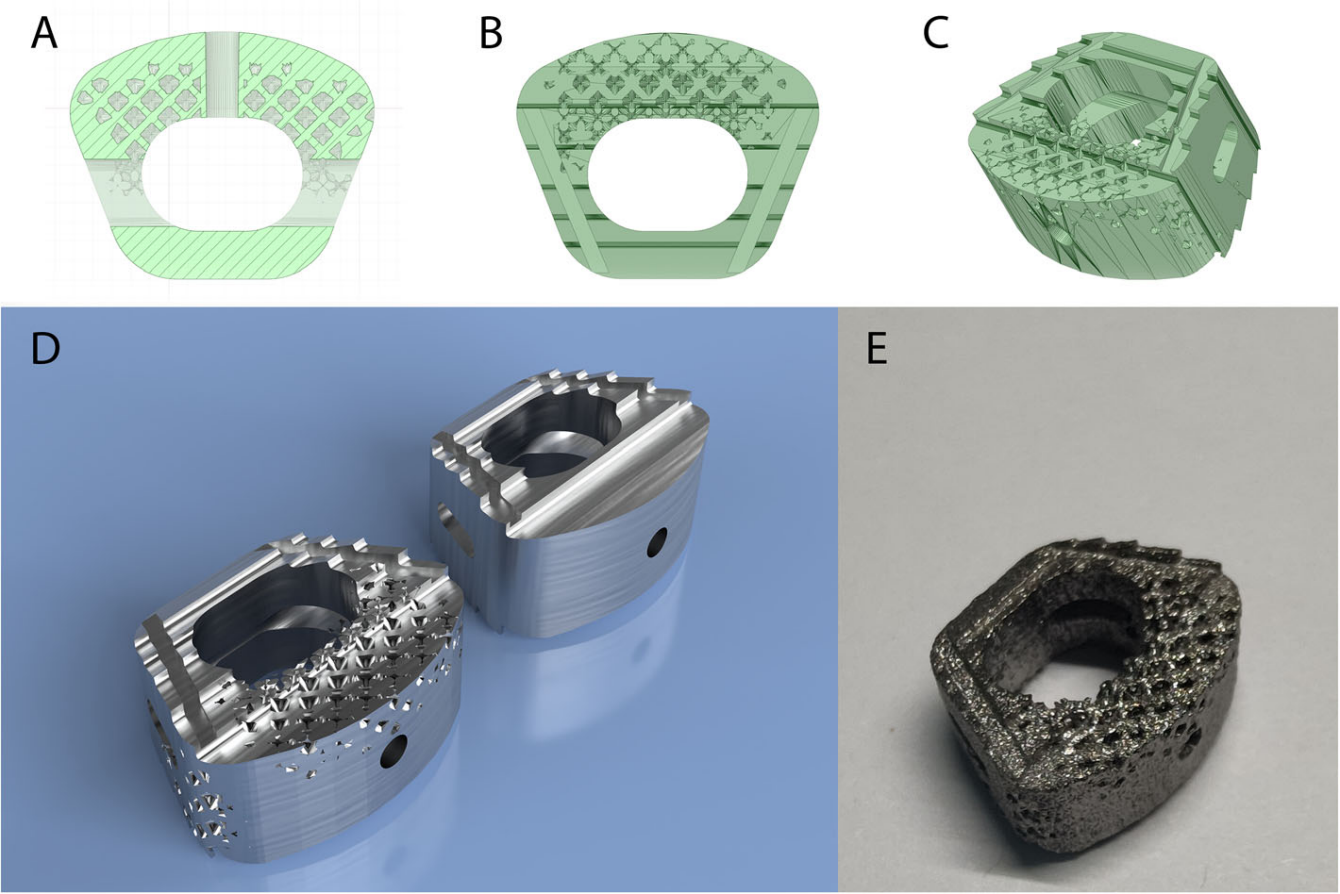
The final model of the optimized fusion cage. **a** The section view of the optimized cage. **b**, **c** Model of the optimized cage. **d** The rendered model of the original and optimized fusion cage. **e** The printed model of the optimized fusion cage

In ANSYS, the lattice structure of the cage can be optimized for additive manufacturing based on the previous period’s analysis data. According to the static structure analysis results, the optimized cloud map is shown in Fig. 11. The original volume of the cage was 766.44mm^3^, while the volume of the optimized structure was 306.58 mm^3^. The porosity was 40%, and the pore structure was Octet, mainly concentrated in the front of the cage. The Geometry and lattice density data of the fusion cage were imported through Spaceclaim software, and Geometry reconstruction was performed through the shell operation (Fig. 12).

### Lattice validation with a homogenization model

By using the homogenization model to analyze each situation, it was found that the cervical spine range of motion was not changed significantly, while the strain of the optimized ZK60 cage was slightly increased. However, no significant difference was observed. The fusion cage’s stress was substantially lower than that of either the ZK60 group or the Ti group (Fig. 8). Simultaneously, the stress of the bone graft was significantly increased compared with the ZK60 and Ti groups (Table 2 and Fig. 9). In contrast, the maximum and average interface stress was decreased considerably (Fig. 10), and the intervertebral disc stress of adjacent segments appeared slightly enhanced. However, no significant difference was observed, as well.

## Discussion

On the premise of satisfying biological safety, the discovery of new biodegradable materials has become an attractive research field in the last few years to overcome the current deficiencies of implants. Titanium alloy fusion cage has been widely used in intervertebral fusion for many years. Due to the high elastic modulus of titanium cages, the fusion rate is relatively high when they are applied for impartment. However, an increased risk of subsidence in titanium cages has been noticed [7, 8].

Magnesium, as an essential macro element for the human body, has a high degree of biocompatibility [13, 14]. Relevant studies have shown that magnesium metal as an implant material does not cause severe inflammation in the body. ZK60, as a potential degradable metal, shows a stiffness close to that of cortical bone, thereby providing better mechanical properties [12–14, 40]. It has been shown in the related calculations that the smaller elastic modulus of the implant material can significantly reduce the stress at the interface between the final plate and the cage, thereby decreasing the subsidence rate [6, 7, 10].

However, few studies have been reported on ZK60 as a fusion cage and a lack of biomechanical experiments. It is unclear whether ZK60 can provide immediate stability while significantly reducing the stress shielding effect. In this study, we utilized the finite element methods to simulate complete intervertebral fusion and to analyze the biomechanical properties of the fusion site and adjacent levels when implanting with different materials’ fusion cages. The biomechanical comparison results of the ZK60 and titanium alloy cages validated the excellent material properties of ZK60. It has been found that although ZK60 cages can effectively reduce the stress shielding effect, the stiffness of traditional cast solid cages is still higher than that of cortical bone. Therefore, we expect that the stiffness of the cage could be further improved through reasonable structural design, thereby making it even closer to the human body. On the other hand, additive manufacturing provides the possibility [41, 42]. Therefore, based on the resulting biomechanical distribution characteristics, we optimized the cage model to generate a porous cage suitable for additive manufacturing. This porous cage has 2-mm-size and heterogeneous lattice structures optimized according to the load. The octet lattice has a structure similar to trabecular bone, which can resist forces in all directions and provide appropriate mechanical support for the new bone [41–45]. At the same time, the pore structure can provide space for bone growth and accelerate intervertebral fusion. The ZK60 material will gradually degrade as time goes by, the stiffness of the cage will gradually decrease, and the mechanical stimulation of new bone will increase progressively. Eventually, the cage will be entirely replaced by the bone structure [21, 22, 40]. The lattice structures are mainly distributed in the front of the cage, while no lattice structure is observed in the rear, as the stress at the rear of the cage is relatively large; the crystal lattice cannot maintain the original mechanical properties; the front stress is small. The distribution of lattice conforms to the features of stress distribution.

The biomechanical properties of the implant are essential to its stability [30, 36]. In our research, no significant difference was observed in all fusion models’ ROMs, which indicates that ZK60 and optimized cages can provide sufficient initial stability to the fusion site while changing the fusion level’s biomechanical properties. A previous study reported that micro-movements greater than 150 μm reduced the interface bonding strength, eventually resulting in implant relaxation [46]. Our results exhibited that the deformation of the ZK60 group was slightly increased under various conditions, while there is no significant difference compared to the Ti group cage. As the maximum displacement of the cage is considered a critical measure of implant stability, the results demonstrate that both ZK60 and titanium alloys’ mechanical properties are good, and both of them can provide immediate stability.

In the stress analysis, the stress of the fusion cage in the optimized group was significantly lower than that in the other two groups under various conditions. Moreover, the stress in the ZK60 group was even smaller than that in the Ti group cage. However, the bone graft stress in the fusion cage shows completely distinct results: the stress of the bone graft in the optimized group was 43.24 and 82.95% greater than that in the ZK60 and Ti groups, respectively. These results may be attributed to the stiffness difference between ZK60 and Ti-6Al-4V, as the elastic modulus of ZK60 is much closer to that of bone compared to titanium alloy. A previous study reported that metal implants with high stiffness could cause stress shielding of the bone surrounding the prosthesis, thereby limiting the load transferred to the bone [47, 48]. Therefore, titanium alloys share part of the load previously withheld merely by bones [49]. According to Wolfe’s law, the structure of the bones is suitable for resisting any force acting on the bones [50], and the bone mass is reduced in response to low stress. Thus, the mismatch in stiffness between the Ti-6Al-4V and bone can lead to stress shielding, resulting in bone resorption and implant loosening. The optimized ZK60 cage further reduces the stiffness through structural optimization and provides the bone graft greater mechanical stimulation while ensuring stability. Relevant studies have shown that mechanical stimulation can significantly increase the speed of bone reconstruction, so a theoretically optimized ZK60 cage has a faster fusion speed than a titanium alloy cage.

It was measured by Grant et al. that the stiffness of different areas on the endplate exhibited a trend of decreasing from the outside to the center of the endplate [51]. Microfractures occur when the local stress is higher than the limit of the relevant area [51–54], leading to osteolysis and cage subsidence [51–54]. Our studies found that the average stress of the ZK60 group and the Ti group were higher by 54.25 and 96.13% compared to that of the optimized group, whose endplate interface stress was only 0.97 MPa on average. We believe that the lower the stiffness of the implant, the smaller the stress on the endplate, the lower possibility of occurrence of microfractures, osteolysis, or cage subsidence. Therefore, the possibility of subsidence of the optimized cage is lower than that of the ZK60 and titanium alloy cage.

A structure with lower stiffness should theoretically maintain a certain degree of movement and reduce the stress on the facet joints and intervertebral discs in adjacent positions [34, 36, 46]. In this study, no significant difference was observed between each group model’s mobility and the adjacent intervertebral disc’s stress, which may be related to the bone graft part of the fusion cage’s stress with a lower elastic modulus. Therefore, the total stress of the overall cage-bone graft system was not changed significantly, which resulted in no significant change in the adjacent intervertebral disc force. In the simulated surgery models, degenerative changes in the facet joints may be correlated, thereby increasing the risk of additional impacts on the biomechanical stability of the cervical spine. In the present study, it was assumed that the height of intervertebral space in the intact and surgery models was identical. Moreover, the bone-cage interface in the surgery models was simplified and considered as well-fused at the bone graft area. Therefore, further studies are still required to investigate these variables.

The present Finite element (FE) study also has several limitations. Firstly, the finite element model without muscles cannot fully imitate the intact cervical spine’s natural state. Secondly, when constructing the finite element model, gender differences and degenerative changes associated with facet degeneration, endplate sclerosis, annular tears, or vertebrae were not considered. Lastly, the ZK60 material used in this experiment is degradable, so the stiffness of the ZK60 cage will be decreased gradually. Overall, the FE analysis can only analyze the biomechanics immediately after the operation, and the stiffness reduction caused by degradation was not taken into account. The mechanical properties of cages after degradation and the control of the degradation rate to match the bone growth status still need further exploration.

## Conclusions

Our findings suggest that using different materials as the cage of the intervertebral fusion has a modest effect on the ROM and adjacent disc stress. However, the application of a ZK60 cage can significantly improve the stress stimulation of bone graft by reducing the stress shielding effect between two instrumented bodies. Furthermore, it has been observed that as the stiffness of the cage decreases, the stress of the endplate-cage interface reduces, which indicates that subsidence is less likely to occur in the cage with lower stiffness. In summary, we’ve successfully designed a porous cage based on the biomechanical load through lattice optimization. The verified results show that an optimized cage can further reduce stiffness than the ZK60 cage, decrease the stress shielding effect, and provide appropriate space for bone growth.

## Data Availability

The datasets generated during and analyzed during the current study are not publicly available due to some data of project is not suitable for disclosure but are available from the corresponding author on reasonable request.

## Abbreviations

FE: Finite element
FEA: Finite element analysis
ACDF: Anterior cervical disc removal and bone graft fusion
ROM: Range of motion
IDD: Intervertebral disc degeneration
PEEK: Polyetheretherketone
CUDA: Compute Unified Device Architecture
CT: Computed tomography

## References

[1] Cloward RB (1958). The anterior approach for removal of ruptured cervical disks. J Neurosurg.

[2] Radcliff K, Coric D, Albert T (2016). Five-year clinical results of cervical total disc replacement compared with anterior discectomy and fusion for treatment of 2-level symptomatic degenerative disc disease: a prospective, randomized, controlled, multicenter investigational device exemption clinical trial. J Neurosurg Spine.

[3] Kim LH, D'Souza M, Ho AL, Pendharkar AV, Sussman ES, Rezaii P, Desai A (2018). Anterior techniques in managing cervical disc disease. Cureus.

[4] Bagby GW (1988). Arthrodesis by the distraction-compression method using a stainless steel implant. Orthopedics.

[5] Scholz M, Schleicher P, Pabst S, Kandziora F (2015). A zero-profile anchored spacer in multilevel cervical anterior interbody fusion: biomechanical comparison to established fixation techniques. Spine.

[6] Yson SC, Sembrano JN, Santos ERG (2017). Comparison of allograft and polyetheretherketone (PEEK) cage subsidence rates in anterior cervical discectomy and fusion (ACDF). J Clin Neurosci.

[7] Schmieder K, Wolzik-Grossmann M, Pechlivanis I, Engelhardt M, Scholz M, Harders A (2006). Subsidence of the wing titanium cage after anterior cervical interbody fusion: 2-year follow-up study. J Neurosurg Spine.

[8] Phan K, Pelletier MH, Rao PJ, Choy WJ, Walsh WR, Mobbs RJ (2019). Integral fixation titanium/polyetheretherketone cages for cervical arthrodesis: evolution of cage design and early radiological outcomes and fusion rates. Orthop Surg.

[9] Nemoto O, Asazuma T, Yato Y, Imabayashi H, Yasuoka H, Fujikawa A (2014). Comparison of fusion rates following transforaminal lumbar interbody fusion using polyetheretherketone cages or titanium cages with transpedicular instrumentation. Eur Spine J.

[10] Le TV, Baaj AA, Dakwar E, Burkett CJ, Murray G, Smith DA, Uribe JS (2012). Subsidence of polyetheretherketone intervertebral cages in minimally invasive lateral retroperitoneal transpsoas lumbar interbody fusion. Spine.

[11] Daentzer D, Willbold E, Kalla K, Bartsch I, Masalha W, Hallbaum M, Hurschler C, Kauth T, Kaltbeitzel D, Hopmann C (2014). Bioabsorbable interbody magnesium-polymer cage: degradation kinetics, biomechanical stiffness, and histological findings from an ovine cervical spine fusion model. Spine.

[12] Brar HS, Platt MO, Sarntinoranont M, Martin PI, Manuel MV (2009). Magnesium as a biodegradable and bioabsorbable material for medical implants. Jom.

[13] Chakraborty Banerjee P, Al-Saadi S, Choudhary L, Harandi SE, Singh R (2019). Magnesium implants: prospects and challenges. Materials.

[14] Liu C, Ren Z, Xu Y, Pang S, Zhao X, Zhao Y (2018). Biodegradable magnesium alloys developed as bone repair materials: a review. Scanning.

[15] Zeng R-C, Qi W-C, Cui H-Z, Zhang F, Li S-Q, Han E-H (2015). In vitro corrosion of as-extruded Mg–Ca alloys—the influence of Ca concentration. Corros Sci.

[16] Xia D, Liu Y, Wang S, Zeng R-C, Liu Y, Zheng Y, Zhou Y (2019). In vitro and in vivo investigation on biodegradable Mg-Li-Ca alloys for bone implant application. Sci China Mater.

[17] Wang H, Guan S, Wang Y, Liu H, Wang H, Wang L, Ren C, Zhu S, Chen K (2011). In vivo degradation behavior of Ca-deficient hydroxyapatite coated Mg–Zn–Ca alloy for bone implant application. Colloids Surf B: Biointerfaces.

[18] Vickers NJ (2017). Animal communication: when i’m calling you, will you answer too?. Curr Biol.

[19] Steinfeld B, Scott J, Vilander G, Marx L, Quirk M, Lindberg J, Koerner K (2015). The role of lean process improvement in implementation of evidence-based practices in behavioral health care. J Behav Health Serv Res.

[20] Jafari H, Rahimi F, Sheikhsofla Z (2016). In vitro corrosion behavior of Mg-5Zn alloy containing low Y contents. Mater Corros.

[21] Qi ZR, Zhang Q, Tan LL, Lin X, Yin Y, Wang XL, Yang K, Wang Y (2014). Comparison of degradation behavior and the associated bone response of ZK60 and PLLA in vivo. J Biomed Mater Res A.

[22] Lin X, Tan L, Wang Q, Zhang G, Zhang B, Yang K (2013). In vivo degradation and tissue compatibility of ZK60 magnesium alloy with micro-arc oxidation coating in a transcortical model. Mater Sci Eng C.

[23] Gu XN, Li N, Zheng YF, Ruan L (2011). In vitro degradation performance and biological response of a Mg–Zn–Zr alloy. Mater Sci Eng B.

[24] Yoganandan N, Kumaresan S, Pintar FA (2001). Biomechanics of the cervical spine Part 2. Cervical spine soft tissue responses and biomechanical modeling. Clin Biomech.

[25] Ganbat D, Kim YH, Kim K, Jin YJ, Park WM (2016). Effect of mechanical loading on heterotopic ossification in cervical total disc replacement: a three-dimensional finite element analysis. Biomech Model Mechanobiol.

[26] Lei Z, Ji X, Li N, Yang J, Zhuang Z, Rottach D (2014). Simulated effects of head movement on contact pressures between headforms and N95 filtering facepiece respirators-part 1: headform model and validation. Ann Occup Hyg.

[27] Zhang QH, Teo EC, Ng HW, Lee VS (2006). Finite element analysis of moment-rotation relationships for human cervical spine. J Biomech.

[28] Kallemeyn N, Gandhi A, Kode S, Shivanna K, Smucker J, Grosland N (2010). Validation of a C2–C7 cervical spine finite element model using specimen-specific flexibility data. Med Eng Phys.

[29] Ha SK (2006). Finite element modeling of multi-level cervical spinal segments (C3–C6) and biomechanical analysis of an elastomer-type prosthetic disc. Med Eng Phys.

[30] Chen W-M, Jin J, Park T, Ryu K-S, Lee S-J (2018). Strain behavior of malaligned cervical spine implanted with metal-on-polyethylene, metal-on-metal, and elastomeric artificial disc prostheses–a finite element analysis. Clin Biomech.

[31] Panjabi MM (2007). Hybrid multidirectional test method to evaluate spinal adjacent-level effects. Clin Biomech.

[32] Liao Z, Fogel GR, Wei N, Gu H, Liu W (2015). Biomechanics of artificial disc replacements adjacent to a 2-level fusion in 4-level hybrid constructs: an in vitro investigation. Med Sci Monit.

[33] Gandhi AA, Kode S, DeVries NA, Grosland NM, Smucker JD, Fredericks DC (2015). Biomechanical analysis of cervical disc replacement and fusion using single level, two level, and hybrid constructs. Spine.

[34] Panjabi MM, Crisco JJ, Vasavada A, Oda T, Cholewicki J, Nibu K, Shin E (2001). Mechanical properties of the human cervical spine as shown by three-dimensional load–displacement curves. Spine.

[35] Liu Q, Guo Q, Yang J, Zhang P, Xu T, Cheng X, Chen J, Guan H, Ni B (2016). Subaxial cervical intradiscal pressure and segmental kinematics following atlantoaxial fixation in different angles. World Neurosurg.

[36] Lee JH, Park WM, Kim YH, Jahng T-A (2016). A biomechanical analysis of an artificial disc with a shock-absorbing core property by using whole-cervical spine finite element analysis. Spine.

[37] Wen CE, Mabuchi M, Yamada Y, Shimojima K, Chino Y, Asahina T (2001). Processing of biocompatible porous Ti and Mg. Scr Mater.

[38] Wen CE, Yamada Y, Shimojima K, Chino Y, Hosokawa H, Mabuchi M (2004). Compressibility of porous magnesium foam: dependency on porosity and pore size. Mater Lett.

[39] Yazdimamaghani M, Razavi M, Vashaee D, Moharamzadeh K, Boccaccini AR, Tayebi L (2017). Porous magnesium-based scaffolds for tissue engineering. Mater Sci Eng C.

[40] Byun S-H, Lim H-K, Lee S-M, Kim H-E, Kim S-M, Lee J-H (2020). Biodegradable magnesium alloy (ZK60) with a poly (l-lactic)-acid polymer coating for maxillofacial surgery. Metals.

[41] Ling C, Cernicchi A, Gilchrist MD, Cardiff P (2019). Mechanical behaviour of additively-manufactured polymeric octet-truss lattice structures under quasi-static and dynamic compressive loading. Mater Des.

[42] Gangireddy S, Komarasamy M, Faierson EJ, Mishra RS (2019). High strain rate mechanical behavior of Ti-6Al-4V octet lattice structures additively manufactured by selective laser melting (SLM). Mater Sci Eng A.

[43] Tancogne-Dejean T, Spierings AB, Mohr D (2016). Additively-manufactured metallic micro-lattice materials for high specific energy absorption under static and dynamic loading. Acta Mater.

[44] Dong L, Deshpande V, Wadley H (2015). Mechanical response of Ti–6Al–4V octet-truss lattice structures. Int J Solids Struct.

[45] Deshpande VS, Fleck NA, Ashby MF (2001). Effective properties of the octet-truss lattice material. J Mech Phys Solids.

[46] Simon U, Augat P, Ignatius A, Claes L (2003). Influence of the stiffness of bone defect implants on the mechanical conditions at the interface—a finite element analysis with contact. J Biomech.

[47] Zhang Q-H, Cossey A, Tong J (2016). Stress shielding in periprosthetic bone following a total knee replacement: effects of implant material, design and alignment. Med Eng Phys.

[48] Chuah HG, Rahim IA, Yusof MI (2010). Topology optimisation of spinal interbody cage for reducing stress shielding effect. Comput Methods Biomech Biomed Eng.

[49] Mi ZR, Shuib S, Hassan AY, Shorki AA, Ibrahim MNM (2007). Problem of stress shielding and improvement to the hip Implat designs: a review. J Med Sci.

[50] Bugbee WD, Sychterz CJ, Engh CA (1996). Bone remodeling around cementless hip implants. South Med J.

[51] Grant JP, Oxland TR, Dvorak MF (2001). Mapping the structural properties of the lumbosacral vertebral endplates. Spine.

[52] Wang H, Lv B (2018). Comparison of clinical and radiographic results between posterior pedicle-based dynamic stabilization and posterior lumbar intervertebral fusion for lumbar degenerative disease: a 2-year retrospective study. World Neurosurg.

[53] Schlegel K-F, Pon A (1985). The biomechanics of posterior lumbar interbody fusion (PLIF) in spondylolisthesis. Clin Orthop Relat Res.

[54] Goulet JA, Senunas LE, DeSilva GL, Greenfield MLVH (1997). Autogenous iliac crest bone graft: complications and functional assessment. Clin Orthop Relat Res.

